# Efficacy, safety, pharmacokinetics and biomarker findings in patients with HER2-positive advanced or metastatic breast cancer treated with lapatinib in combination with capecitabine: results from 51 Japanese patients treated in a clinical study

**DOI:** 10.1007/s12282-013-0475-1

**Published:** 2013-05-21

**Authors:** Hiroji Iwata, Hirofumi Fujii, Norikazu Masuda, Hirofumi Mukai, Yuichiro Nishimura, Koichi Katsura, Catherine E. Ellis, Robert C. Gagnon, Seigo Nakamura

**Affiliations:** 1 Aichi Cancer Center Hospital, Breast Oncology, 1-1 Kanokoden, Chikusa-ku, Nagoya, Aichi 464-8681 Japan; 2 Jichi Medical University Hospital, Tochigi, Japan; 3 National Hospital Organization Osaka National Hospital, Osaka, Japan; 4 National Cancer Center Hospital East, Chiba, Japan; 5 GlaxoSmithKline K.K., Tokyo, Japan; 6 GlaxoSmithKline, Pennsylvania, USA; 7 St. Luke’s International Hospital, Tokyo, Japan; 8 Showa University Hospital, Tokyo, Japan

**Keywords:** Lapatinib, Capecitabine, HER2, Breast cancer, Biomarker

## Abstract

**Background:**

The results from a phase III trial conducted outside of Japan demonstrated a significant improvement in time to progression (TTP) when lapatinib was combined with capecitabine compared with capecitabine alone in patients with HER2-positive advanced or metastatic breast cancer. In this clinical study of lapatinib in combination with capecitabine, efficacy, safety, pharmacokinetics (PK) and biomarkers were investigated in Japanese patients with HER2-positive advanced or metastatic breast cancer treated with prior trastuzumab.

**Methods:**

Eligible women received lapatinib 1250 mg once daily and capecitabine 1000 mg/m^2^ twice daily on days 1 through 14 of a 21-day cycle. The primary endpoint was the clinical benefit rate (CBR: complete response, partial response or stable disease for at least 24 weeks).

**Results:**

Lapatinib in combination with capecitabine was well tolerated in the 51 patients enrolled in this study. CBR was 59 % (95 % CI 44.2, 72.4), and the median TTP in the Kaplan-Meier estimate was 36 weeks (95 % CI 27.1, 48.0). The majority of drug-related adverse events were mild to moderate (grade 1 or 2); the most common adverse events reported were palmar-plantar erythrodysesthesia syndrome (76 %), diarrhea (67 %) and stomatitis (41 %).

**Conclusions:**

Lapatinib in combination with capecitabine in Japanese HER2-positive breast cancer patients was well tolerated. Overall, our findings on the efficacy, safety and PK were similar to those reported from the overseas studies.

## Introduction

Approximately 20 % of breast cancers exhibit overexpression or gene amplification of human epidermal growth factor receptor 2 (HER2), which is known to be associated with aggressive disease and a greater risk of disease progression and death [1].

Lapatinib is a reversible, bioavailable and small molecule tyrosine kinase inhibitor (TKI) that potently inhibits both epidermal growth factor receptor (EGFR) and HER2. EGF receptor family TKIs, such as lapatinib, compete intracellularly with adenosine triphosphate for its receptor binding site and inhibit the tyrosine kinase activity of the receptor. In principle, small molecule TKIs should inhibit the activity of EGF receptors in the presence of elevated levels of ligand as well as the activity of EGF receptors with truncated extracellular domains (ECDs). In fact, single agent small molecule TKIs have been reported to exhibit clinical activity [2].

A randomized, overseas phase III study evaluating the efficacy and safety of lapatinib in combination with capecitabine in women with HER2-positive advanced or metastatic breast cancer (MBC) that progressed following prior therapy including trastuzumab provided the evidence of the efficacy and safety for this combination [3–5]. Further studies evaluating lapatinib and capecitabine have been reported in the past [6–9]; however, these studies were not conducted in Japan. We now report the findings from a clinical study of the safety, efficacy, pharmacokinetics (PK) and biomarkers in Japanese patients with HER2-positive advanced or MBC treated with lapatinib in combination with capecitabine.

## Patients and methods

### Study design

This was an open-label, multicenter study. The objectives of this study were to evaluate the safety, tolerability, PK and efficacy as determined by the clinical benefit rate [CBR: complete response (CR), partial response (PR) or stable disease (SD) for at least 24 weeks] of lapatinib in combination with capecitabine at the overseas recommended dosage (i.e., lapatinib 1250 mg/day QD and capecitabine 2000 mg/m^2^/day BID) in Japanese patients with HER2-positive advanced or MBC. Secondary endpoints were time to progression (TTP: the interval between the start of treatment and the earliest date of disease progression or death due to breast cancer, whichever is sooner), progression-free survival (PFS), 6-month progression-free survival, overall response rate [ORR: the percentage of subjects achieving best overall response classified as CR or PR (confirmed)], overall survival (OS), time to response, duration of response, PK parameters and adverse events (throughout the study period).

The study consisted of two parts; the first part (part 1) was to determine the safety, tolerability and PK parameters of lapatinib given with capecitabine in the six patients enrolled. This was done as it was the first time Japanese patients had received lapatinib in combination with capecitabine. If no major safety concerns were observed in part 1, the study moved into the second part (part 2) to further evaluate safety as well as efficacy and biomarkers.

The tolerability criteria in part 1 were defined as grade 3 or 4 drug-related non-hematological toxicity (excluding ≥ grade 3 diarrhea, nausea or vomiting); grade 3 or 4 drug-related diarrhea, nausea or vomiting in the presence of supportive care; hematological toxicity (grade 4 drug-related neutropenia lasting ≥ 5 days, febrile neutropenia ≥ grade 3 or thrombocytopenia ≤ 25000/mm^3^); inability to start the next treatment cycle due to unresolved toxicity; missing > 50 % of scheduled doses in a cycle due to toxicity. If two or fewer subjects in the six subjects experienced a safety issue corresponding with the tolerability criteria, the study medication was concluded to be the tolerable regimen. The study was conducted in accordance with Good Clinical Practice guidelines and the Declaration of Helsinki.

### Patient eligibility

Eligible patients were women aged 20 years or older with histologically confirmed invasive and advanced or metastatic (stage IIIB, stage IIIC with T4 lesion or stage IV) breast cancer that was HER2-positive [confirmed immunohistochemistry (IHC) 3 + or IHC 2 +/fluorescence in situ hybridization (FISH) positive] by a local laboratory. Patients were required to have measurable lesion(s) according to the Response Evaluation Criteria in Solid Tumors (RECIST) version 1.0 [10]. Patients must have received prior therapy including anthracyclines, taxanes and trastuzumab. Additional inclusion criteria were Eastern Cooperative Oncology Group (ECOG) performance status of 0 or 1, life expectancy of at least 12 weeks, left ventricular ejection fraction (LVEF) within the institutional normal range (or ≥ 50 %), adequate renal, hepatic and hematologic functions, and archived tumor tissue available for biomarker analysis. Patients were excluded if they had had prior therapy with capecitabine or an EGFR and/or HER2 inhibitor other than trastuzumab, or if they had malabsorption syndrome or other conditions that would prevent the efficacy and safety evaluation of study regimen. All patients signed an informed consent.

### Treatment schedule

Lapatinib 1250 mg was taken orally once daily at approximately the same time every day. Capecitabine was taken orally twice daily (needed 12-h of administration interval) for 14 days in every 21 days. Each dose of capecitabine was 1000 mg/m^2^. Subjects were treated until disease progression or unacceptable toxicity.

### Safety and efficacy assessments

Safety assessment was performed every week for the first 3 weeks, every 6 weeks from week 3 to week 24, then every 12 weeks and at the end of treatment. Adverse events were checked and laboratory tests were performed in all subjects every 3 weeks. LVEF assessment by echocardiogram and efficacy assessments were performed every 6 weeks for 24 weeks, then every 12 weeks and at the end of treatment. Subjects withdrawn from the study without disease progression were assessed every 12 weeks until progression, start of post anticancer therapy or death. Adverse events were assessed according to the National Cancer Institute Common Terminology Criteria for Adverse Events (CTCAE version 3.0). Efficacy was assessed by the independent review using images or photographic data, in accordance with the RECIST. During the study treatment, investigators reviewed tumor response for timely evaluations.

### PK assessment

PK assessment was performed in six subjects enrolled into part 1: blood samples were obtained on day 14 for over 24-h period. Blood samples [2 ml was collected in ethylenediaminetetraacetic acid (EDTA) tubes] for the analysis of lapatinib were drawn before dosing and 1, 2, 3, 4, 6, 8, 10, 12 and 24 h after dosing of lapatinib. Blood samples (4 ml, collection in EDTA tubes) for the analysis of capecitabine, 5-fluorouracil (5-FU) and α-fluoro-β-alanine (FBAL) were drawn before dosing and 0.5, 1, 2, 3, 4, 6, 8 and 10 h after the morning dose of capecitabine.

PK samples were also collected from the subjects enrolled in part 2 to determine the plasma drug concentrations on day 14 and day 21: blood samples (2 ml for lapatinib and 6 ml for capecitabine, 5-FU and FBAL) were taken at two time points: before lapatinib and the morning dose of capecitabine on day 14 (for assay of lapatinib, capecitabine, 5-FU and FBAL) and before dosing of lapatinib on day 21 (for assay of lapatinib). Blood plasma was cold centrifuged at about 1000 × *g* for 10–15 min, and the plasma gained was stored at −20 °C or lower.

PK parameters were calculated using WinNonlin Professional version 4.1. In part 1, maximum plasma concentration (*C*_max_), time to *C*_max_ (*t*_max_), terminal elimination half-life (*t*_1/2_), area under the plasma concentration–time curve within the dosing interval (AUC_0–τ_) and area under the plasma concentration–time curve from 0 to 24 h (AUC_0–24_) of lapatinib, *C*_max_, *t*_max_, *t*_1/2_, AUC_0–τ_ and area under the plasma concentration–time curve from 0 to 12 h (AUC_0–12_) of capecitabine, 5-FU and FBAL were calculated as the PK parameters. In part 2 trough concentrations of lapatinib, capecitabine, 5-FU and FBAL were measured.

### Statistical analysis

This study was designed to perform no hypothesis testing. A sample size was determined based on the study feasibility. An intent-to-treat (ITT) population was used for efficacy data analyses. For subjects who had withdrawn from the study regimen without disease progression or death, TTP was censored at the day of evaluation just before initiating an alternative anticancer therapy. Version 9.2.3 Windows SAS^®^ System (SAS is a registered trademark of the SAS Institute, Inc., Cary, NC, USA) was used to analyze the data.

### Biomarker assessments

Formalin-fixed, paraffin-embedded (FFPE) archived tumor tissue blocks (or sections) from time of original diagnosis or from recurrent/metastatic site were required for enrollment. Intratumoral EGFR protein expression levels were determined using the EGFR pharmDx™ assay (Dako).

Serum blood samples were collected at baseline for the quantitative determination of serum HER2 ECD levels using HER-2/neu enzyme-linked immunosorbent assay (Wilex, Inc.).

HER2 ECD analyses applied a pre-specified cutoff value of 15 ng/ml whereby subjects were grouped according to whether HER2 ECD levels were elevated (> 15 ng/ml) or at the reference normal value of 15 ng/ml or below. Additionally, subjects were grouped according to EGFR IHC scores: IHC 0 was considered as EGFR negative and IHC 1 +/2 +/3 + considered as EGFR positive. TTP was summarized using Kaplan-Meier curves and compared between subgroups using a stratified log-rank test. For CBR, HER2 ECD subgroups were compared using the Wilcoxon rank sum test. Statistical tests with an alpha level of 5 % were considered to indicate statistical significance. Analyses were conducted using SAS version 9.1.3.

## Results

### Patient characteristics

During the period from June 2007 to September 2008, a total of 51 patients were enrolled from 15 centers and treated with the study regimen. As of the final data collection date, 46 subjects (90 %) had discontinued the treatment because of disease progression and 5 (10 %) because of adverse events. Overall, 36 deaths (71 %) were reported.

The majority of patients (80 %) had histologically diagnosed invasive carcinoma NOS (Table 1). Most subjects (71 %) had estrogen receptor (ER)-negative breast cancer as assessed by a local laboratory. The majority of patients had two or more metastatic sites at screening. All patients had at least three prior anti-tumor regimens including anthracyclines, taxanes and trastuzumab. Most patients (78 %) had received their last trastuzumab treatment less than 8 weeks prior to study enrollment.

**Table 1:** Baseline characteristics of ITT population

Age, years	
Median (range)	55.0 (35-75)
ECOG PS, *n* (%)	
0	39 (76)
1	12 (24)
2	0
Disease type, *n* (%)	
Invasive carcinoma NOS	41 (80)
Papillary tubular carcinoma	9 (18)
Invasive lobular carcinoma	1 (2)
Time since diagnosis (months) (*n* = 44)	
Minimum	10
1st quartile	24.5
Median	45.6
3rd quartile	68.2
Maximum	137
Disease stage at the initial diagnosis, *n* (%)	
I	1 (2)
II	20 (39)
III	21 (41)
IV	6 (12)
Unknown	3 (6)
Prior anti-cancer therapy, *n* (%)	
Chemotherapy	51 (100)
Anthracyclines	51 (100)
Taxanes	51 (100)
Trastuzumab	51 (100)
Surgery	46 (90)
Radiotherapy	28 (55)
Endocrine therapy	20 (39)
Vaccines	0
Immunotherapy	0
Duration from completion of trastuzumab, *n* (%) (weeks)	
<8	40 (78)
≥8	11 (22)
Number of metastatic sites, *n* (%)	
≥3	21 (41)
2	19 (37)
1	11 (22)
Visceral or nonvisceral metastatic, *n* (%)	
Visceral	42 (82)
Nonvisceral	9 (18)
Hormone receptor status, *n* (%)	
ER+ and/or PgR+	15 (29)
ER+ and PgR+	7 (14)
ER+ and PgR−	8 (16)
ER− and PgR−	35 (69)
Unknown	1 (2)

*ER* estrogen receptor, *PgR* progesterone receptor

The median duration of lapatinib treatment was 182 days and that of capecitabine was 128 days. The longest treatment period of lapatinib was 833 days.

### Tolerability and safety

In part 1, the safety and tolerability of study treatment were assessed in six subjects. During two cycles when tolerability was evaluated according to the protocol, all subjects experienced drug-related adverse events, such as fatigue, diarrhea and pruritus. All adverse events were grade 1 or 2 in severity with the exception of one case of a grade 3 neutrophil count decrease. This subject also experienced grade 2 cystitis and a grade 2 white blood cell (WBC) count decrease. None of the subjects were considered to have met the tolerability criteria. Based on these findings, the combination of lapatinib with capecitabine established in earlier studies was also tolerable in Japanese MBC patients.

A total of 51 subjects experienced at least one adverse event regardless of the relationship with the study treatments, and most of which were at grade 1 or 2. The most common adverse events reported were palmar-plantar erythrodysesthesia (PPE) syndrome, diarrhea and stomatitis (Table 2). In addition, all subjects experienced drug-related adverse events during the study periods. Grade 4 serious adverse events reported were an alanine aminotransferase increase, neutrophil count decrease, bone marrow failure and pericardial effusion.

**Table 2:** Summary of adverse events experienced by at least 10 % of 51 subjects

Adverse event, *n* (%)	Grade 1	Grade 2	Grade 3	Grade 4	Total
PPE syndrome	18 (35)	16 (31)	5 (10)	0	39 (76)
Diarrhea	26 (51)	7 (14)	1 (2)	0	34 (67)
Stomatitis	21 (41)	0	0	0	21 (41)
Rash	13 (25)	6 (12)	1 (2)	0	20 (39)
Pruritus	16 (31)	1 (2)	0	0	17 (33)
Nausea	15 (29)	2 (4)	0	0	17 (33)
Fatigue	16 (31)	1 (2)	0	0	17 (33)
Anorexia	15 (29)	1 (2)	1 (2)	0	17 (33)
Blood bilirubin increased	6 (12)	10 (20)	0	0	16 (31)
Dry skin	13 (25)	1 (2)	1 (2)	0	15 (29)
Alanine aminotransferase increased	9 (18)	4 (8)	1 (2)	1 (2)	15 (29)
Aspartate aminotransferase increased	9 (18)	3 (6)	3 (6)	0	15 (29)
Nasopharyngitis	14 (27)	1 (2)	0	0	15 (29)
White blood cell count decreased	2 (4)	10 (20)	2 (4)	0	14 (27)
Paronychia	11 (22)	3 (6)	0	0	14 (27)
Neutrophil count decreased	3 (6)	4 (8)	3 (6)	1 (2)	11 (22)
Pigmentation disorder	9 (18)	0	0	0	9 (18)
Pyrexia	8 (16)	1 (2)	0	0	9 (18)
Blood alkaline phosphatase increased	7 (14)	0	1 (2)	0	8 (16)
Red blood cell count decreased	8 (16)	0	0	0	8 (16)
Cheilitis	7 (14)	0	0	0	7 (14)
Weight decreased	4 (8)	3 (6)	0	0	7 (14)
Headache	6 (12)	1 (2)	0	0	7 (14)
Skin exfoliation	6 (12)	1 (2)	0	0	7 (14)
Vomiting	4 (8)	2 (4)	0	0	6 (12)
Malaise	5 (10)	1 (2)	0	0	6 (12)
Dizziness	5 (10)	0	1 (2)	0	6 (12)
Cancer pain	3 (6)	3 (6)	0	0	6 (12)
Acne	3 (6)	1 (2)	1 (2)	0	5 (10)
Nail disorder	4 (8)	1 (2)	0	0	5 (10)
Constipation	5 (10)	0	0	0	5 (10)
Blood albumin decreased	3 (6)	2 (4)	0	0	5 (10)

Number (percent)

*PPE* palmar-plantar erythrodysesthesia

A total of two fatal serious adverse events (dysphagia and respiratory failure) were reported in one subject and determined to be unrelated to the study medication by the investigator. Disease progression was considered to be the major cause of death. Eight additional serious adverse events were reported in seven subjects (14 %). These included five treatment-related serious adverse events that occurred in four subjects: grade 3 vertigo and grade 3 syncope were observed in one subject, grade 1 and 2 left ventricular dysfunction were observed in one subject each, and grade 2 pulmonary tuberculosis was observed in one subject. Cardiac events, which are known to be a potential risk of HER2 target agents including lapatinib and trastuzumab, occurred in three subjects (6 %). These subjects experienced left ventricular dysfunction that was asymptomatic and later resolved.

### Pharmacokinetics

In part 1, following administration of lapatinib in combination with capecitabine on day 14 (*n* = 5), *t*_max_ values were approximately 3–6 h for lapatinib, 0.5–3 h for capecitabine, approximately 2 h for 5-FU and approximately 3 h for FBAL (Table 3). Co-administered lapatinib and capecitabine were rapidly absorbed, and plasma concentrations of lapatinib, capecitabine, 5-FU and FBAL showed rapid increases. Plasma concentrations of lapatinib, capecitabine and 5-FU reached *C*_max_ and then decreased in a linear fashion. *t*_1/2_ values of FBAL were just under 2.5 h, which was longer than that of capecitabine and 5-FU by approximately three-fold.

**Table 3:** Pharmacokinetic parameters of five subjects

	AUC_0–τ_^a^ (μg h/ml)	*C*_max_^a^ (μg/ml)	*t*_max_^b^ (h)
Lapatinib	48.2 (34.6, 67.1)	3.52 (2.57, 4.82)	5.55 (3.02, 5.93)
Capecitabine	4.00 (3.01, 5.31)	2.70 (1.49, 4.88)	1.90 (0.53, 3.25)
5-FU	0.51 (0.35, 0.76)	0.28 (0.13, 0.63)	1.90 (0.53, 3.25)
FBAL	29.0 (26.4, 32.0)	5.77 (5.00, 6.66)	3.05 (2.00, 4.25)

^a^Geometric mean (95 % confidence interval)

^b^Median (range)

In part 2, mean (standard deviation) pre-dose concentrations of lapatinib were 1.07 (0.72) μg/ml on day 14 and 1.24 (0.85) μg/ml on day 21. At pre-dose on day 14, plasma concentrations of capecitabine were below the limit of quantitation in most of the subjects, plasma concentrations of 5-FU were below the limit of quantitation in all of the subjects, and the mean (standard deviation) plasma concentration of FBAL was 0.67 (0.46) μg/ml.

### Efficacy

**Fig. 1:**
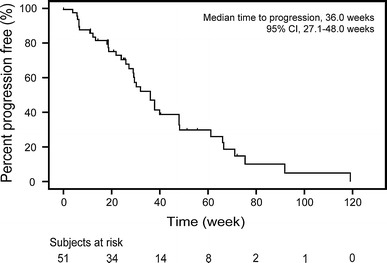
Kaplan-Meier estimates for the time to progression as assessed at the Independent Review Facility

The primary endpoint of this study was CBR. Of 51 patients enrolled, 30 subjects (59 %) derived clinical benefit (95 % CI 44.2, 72.4) (Table 4). ORR in the ITT population was 24 % (95 % CI 12.8, 37.5) with 12 PRs (24 %), while SD and progressive disease (PD) were observed in 63 % (32/51) and 12 % (6/51), respectively; one subject had an unknown response. The median TTP was 36.0 weeks (95 % CI 27.1, 48.0) (Fig. 1). The median OS was 78.6 weeks (95 % CI 51.6, 103.0).

**Table 4:** Summary of tumor response in the ITT population

Best response, *n* (%)	
CR	0
PR	12 (24)
SD, ≥ 24 weeks	18 (35)
SD, < 24 weeks	14 (27)
PD	6 (12)
NE	1 (2)
ORR	24 % (95 %CI 12.8, 37.5)
CBR	59 % (95 %CI 44.2, 72.4)

*CR* complete response, *PR* partial response, *SD* stable disease, *PD* progressive disease, *NE* not evaluable, *ORR* overall response rate, *CBR* clinical benefit rate (CR; PR; SD ≥ 24 weeks)

### Biomarker

All subjects enrolled were HER2-positive (IHC 3 + or FISH positive) by a local laboratory. Based on results of the HER2 status assessed at the central laboratory, 4 % (2/50) were IHC 0, 4 % (2/50) 1 + , 32 % (16/50) 2 +  and 60 % (30/50) 3 + ; 91 % (43/47) were FISH positive (ratio ≥ 2.0) and 9 % (4/47) were negative (ratio < 2.0) (Table 5). Three out of 47 subjects were evaluated as HER2 negative by IHC and FISH, i.e. one subject was IHC 0/FISH negative, another was IHC 1 +/FISH negative, and the other was IHC 2 +/FISH negative. There was one subject whose tumor tissue was non-evaluable for HER2 testing.

**Table 5:** Distribution of HER2 and EGFR biomarker results

HER2 IHC score, *n* (%)	(*n* = 50)
0	2 (4)
1+	2 (4)
2+	16 (32)
3+	30 (60)
FISH HER2 amplification, *n* (%)	(*n* = 47)
Positive (≥ 2.0)	43 (91)
Borderline (1.8 to < 2.0)	0
Negative (< 1.8)	4 (9)
EGFR IHC score, *n* (%)	(*n* = 49)
0	34 (69)
1+	4 (8)
2+	10 (20)
3+	1 (2)

HER2 eligibility was determined at the local laboratory. One subject was non-evaluable for HER2 and EGFR

*HER2* human epidermal growth factor receptor 2, *IHC* immunohistochemistry, *FISH* fluorescence in situ hybridization, *EGFR* epidermal growth factor receptor

Of 49 subjects with EGFR status assessed centrally by IHC, 69 % (34/49) were IHC 0, 8 % (4/49) 1 + , 20 % (10/49) 2 + and 2 % (1/49) 3 +; thus, the majority of subjects had tumors that were EGFR negative.

For exploratory purposes, subgroup analysis was performed in terms of the HER2 status evaluated in the central laboratory (Table 6). In terms of HER2 status evaluated by IHC and FISH, as expected, ORR and CBR were higher in the HER2-positive subgroup, althougha small number of subjects had IHC or FISH negative tumors.

**Table 6:** Subgroup analysis of centrally evaluated EGFR/HER2 status

EGFR IHC	IHC 0 (*n* = 34)	IHC 1 + (*n* = 4)	IHC 2 + (*n* = 10)	IHC 3 + (*n* = 1)
ORR (%) (95 % CI)	29 (14, 44)	0	20 (0, 45)	0

HER2 IHC	IHC 0 (*n* = 2)	IHC 1 + (*n* = 2)	IHC 2 + (*n* = 16)	IHC 3 + (*n* = 30)
ORR (%) (95 % CI)	0	0	13 (2, 38)	33 (17, 53)

HER2 FISH	FISH negative (*n* = 4)	FISH positive (*n* = 43)		
ORR (%) (95 % CI)	0	28 (15, 44)		

*EGFR* epidermal growth factor receptor, *FISH* fluorescence in situ hybridization, *HER2* human epidermal growth factor receptor 2, *IHC* immunohistochemistry, *ORR* overall response rate (CR; PR)

ORR was similar in EGFR IHC 0 and 2 + subgroups; there was a possible trend of a correlation between EGFR status and tumor response; however, inference testing was not performed. There was no significant difference in TTP between EGFR-negative (IHC 0) and -positive (IHC 1 + , 2 + , 3 +) subgroups (*p* = 0.8025).

**Fig. 2:**
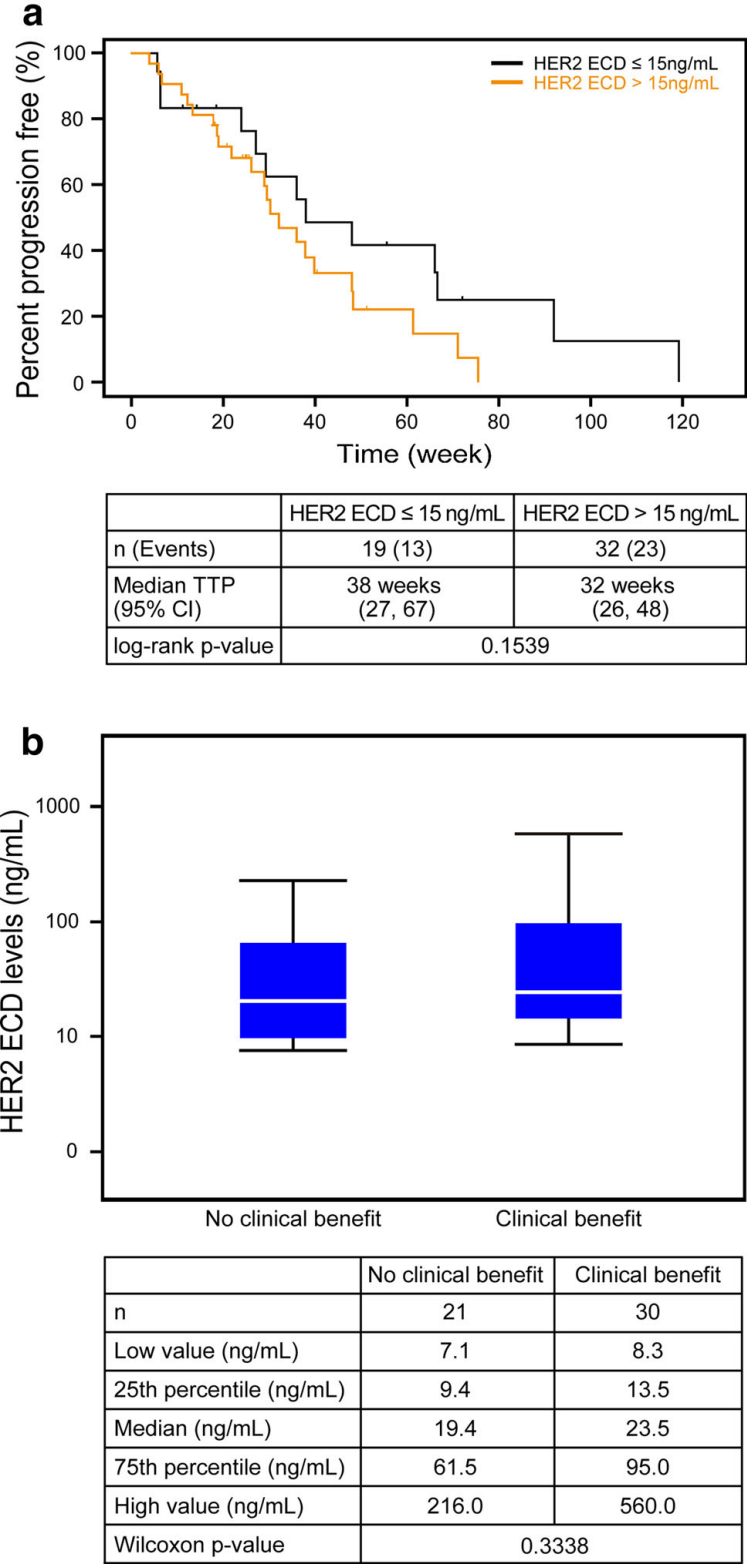
Baseline serum concentration of HER2 ECD according to response to lapatinib plus capecitabine. **a** Kaplan-Meier plots stratified by HER2 ECD status; **b** box plot of HER2 ECD level according to the clinical benefit

Baseline serum HER2 ECD concentration levels (collected prior to this study treatment) were available for all subjects. At screening, the majority of subjects had HER2 ECD levels > 15 ng/ml (63 %). This finding may be reflective of the advanced disease stage and overall tumor burden of the population [11]. Exploratory analysis showed no significant difference in TTP between two HER2 ECD subgroups and no significant difference in HER2 ECD levels between the no clinical benefit group and clinical benefit group (*p* = 0.1539 and 0.3338, respectively) (Fig. 2a, b).

## Discussion

In this study, lapatinib in combination with capecitabine was well tolerated in Japanese patients with HER2-positive MBC who had been treated with prior anthracyclines, taxanes and trastuzumab. The safety results from 51 Japanese subjects indicated adverse events were manageable and consistent with the safety profile previously reported [3, 4].

A phase I study, conducted outside of Japan, investigated the drug-drug interaction between capecitabine and lapatinib [6]. The results suggested there were no significant changes in the PK of lapatinib, capecitabine and its metabolites, 5-FU and FBAL. The 90 % confidence interval for PK parameters, obtained from overseas study of 19 subjects, the AUC and *C*_max_ of capecitabine were 5.64, 7.98 and 4.31, 7.98, respectively, whereas those of FBAL were 16.3, 25.5 and 3.38, 4.65. The capecitabine intervals were lower, whereas the FBAL intervals were higher in Japanese subjects compared with foreign subjects. However, no differences were found (overlapping of confidence intervals) in the AUC and *C*_max_ of another capecitabine metabolite, 5-FU (90 % CI 0.520, 0.772), and of lapatinib (90 % CI 32.7, 63.2). Furthermore, the median *t*_max_ for lapatinib, capecitabine, 5-FU and FBAL was similar between Japanese and foreign subjects. These findings suggest there are no clinically significant differences between the two populations.

The pivotal overseas study reported that lapatinib in combination with capecitabine would be a recommended treatment option for patients with HER2-positive MBC who had been previously treated with therapy including trastuzumab [3, 4]. In this study report, the median TTP in Japanese subjects was 36.0 weeks (95 %CI 27.1, 48.0), which is comparable to overseas results (27.1 weeks). The comparisons of TTP indicate a consistency between the two study populations without the possibility of an ethnic difference.

The tumor response rate in this study (24 %) also indicated consistency with overseas studies (24 %). In contrast, CBR in this study (59 %) was higher than that in overseas studies (29 %). The tumor response evaluations by the independent review were conducted in the same independent facility for both studies; therefore, practical bias can be excluded. The higher CBR compared with the overseas study is considered to be related with the higher frequency of the patients who have achieved ≥ 24 weeks SD. This finding is consistent with the results from another report by Toi et al. [12] that reported a higher CBR in a Japanese population treated with lapatinib monotherapy. By making comparisons with the previously reported overseas study, there were no clear differences in tumor profiles at baseline such as HER2 status or adverse events observed during treatment, except the discrepancy in hormone receptor status, which was observed in the patient’s background (ER- and PgR- rate; overseas study 48 % vs. this study 69 %). This study has limitations in providing details for explaining how this discrepancy can be reasoned. One of the possibilities is, as previously reported, that breast cancer is a heterogeneous disease, and, in particular, HER2-positive breast cancer is known to have variable biological features based upon gene expression profiling, such as luminal HER2+ tumors, HER2-enriched and basal HER2+ tumors [13–17].

From this study on lapatinib and capecitabine given in a Japanese population, a correlation between the HER2 IHC score and tumor response has been suggested. This finding is similar to that reported by Toi et al. [12]. No clear correlation was observed between EGFR expression and tumor response; this finding is consistent with that previously reported by Finn et al., suggesting no correlation with EGFR expression and a benefit from lapatinib with paclitaxel [18]. There was no significant difference in TTP and CBR observed between the two HER2 ECD subgroups. However, no suggestion can be derived from this finding as the study population was too small.

The efficacy and safety outcome of this study were consistent with previous reports on lapatinib in combination with capecitabine. These findings confirm that this regimen offers a clinically beneficial therapeutic option for HER2-positive breast cancer patients in Japan.

Most recently, the results from clinical trials of the new HER2-targeting agents pertuzumab and trastuzumab emtansine have been reported, and these agents can be considered as options in the HER2-positive breast cancer treatment algorithm [19, 20]. As stated in this and previous reports, the lapatinib and capecitabine combination shows anti-tumor efficacy even in patients who have undergone heavy treatment. Thus, lapatinib and capecitabine should still be options for long-term treatment of breast cancer. In order to maximize the treatment options for HER2-positive breast cancer patients, the suitable clinical position of the lapatinib and capecitabine combination needs to be clarified by further evaluations including biomarkers.
